# Ethnoveterinary of Sahrawi pastoralists of Western Sahara: camel diseases and remedies

**DOI:** 10.1186/s13002-015-0040-4

**Published:** 2015-06-20

**Authors:** Gabriele Volpato, Saleh Mohamed Lamin Saleh, Antonello Di Nardo

**Affiliations:** Center for Integrative Conservation Research (CICR), University of Georgia, Athens, GA USA; Sahrawi Veterinary Services, Ministry of Public Health, Sahrawi Arab Democratic Republic, Rabouni, Algeria; Institute of Biodiversity, Animal Health and Comparative Medicine, College of Medical, Veterinary and Life Sciences, University of Glasgow, Glasgow, UK; The Pirbright Institute, Pirbright, Woking, Surrey UK

**Keywords:** Sahrawi refugees, Camels, Western Sahara, Pastoralism, Ethnoveterinary, Camel diseases, Traditional remedies

## Abstract

**Background:**

Pastoral populations around the world hold complex and detailed ethnoveterinary knowledge, essential for the survival of their herds and securing their livelihood. In recent decades, several studies have given attention to local veterinary remedies and practices and their validation, and to the local conceptualization of livestock diseases. Despite this, relatively little has been reported on indigenous knowledge of camel diseases (e.g., aetiological factors, epidemiological patterns, symptoms, prevention and treatments). This paper focuses on the traditional knowledge of camel diseases and their treatments among Sahrawi nomads, detailing how this knowledge is powerfully reflected on pastoral adaptation strategies to the ecological system of Western Sahara.

**Methods:**

Between 2005 and 2010, freelisting exercise on camel diseases with 46 Sahrawi nomads and refugees, semi-structured interviews with 36 knowledgeable informants about camel diseases and associated treatments, and a voucher specimen collection of the plants and products cited were conducted in the territories administered by the Saharawi Arab Democratic Republic, Western Sahara. Analytical methods included standard ethnobiological, ethnobotanical and cultural consensus analyses.

**Results:**

In total, 42 camel diseases were freelisted by informants, with four (i.e., mange, dermatomycosis, respiratory infections, and mastitis) found to be culturally highly salient. These four represent the most common veterinary conditions experienced by Sahrawi pastoralists. In addition, 42 plant species belonging to 22 botanical families (*Hammada scoparia*, *Acacia tortilis*, *Zygophyllum gaetulum*, *Nucularia perrinii*, and *Panicum turgidum* among the species most used) were listed as used in the treatment of these diseases, as well as about 30 remedies of animal (e.g., from camels, donkeys, and/or spiny-tailed lizards) and mineral origin, and of cauterizations.

**Conclusions:**

This study provides an overall picture of the most important camel diseases and remedies as reported by Sahrawi informants, detailing how the vast knowledge that the Sahrawi hold on the health and disease of their camels is constructed through contrasts between their customary nomadic land (and associated climate, soils, grazing and therapeutic resources) and the surrounding areas (and associated diseases), which are traditionally used only in cases of drought.

## Background

Ethnoveterinary medicine is the holistic and interdisciplinary study of the knowledge and practices of indigenous populations in relation to the management of animals used with different purposes, along with the social structure in which these are embedded [1, 2]. During the last decades the study and application of ethnoveterinary medicine have been increasing [3], contributing to several aspects of the scientific research and its application on animal health (e.g., health and nutrition, conservation and management of livestock, natural resources, and ecosystems) [4–6]. Recently, several studies have given attention to local veterinary remedies and practices and their validation [7–9], and to the local conceptualization of livestock illnesses [10, 11]. However, a gap in the knowledge about camels still exists, especially on patterns, causes and effects of diseases impacting on nomadic management systems [12]. Although camels are usually depicted as extremely resistant animals, they are nevertheless often subjected to severe stress conditions which make them susceptible to a range of veterinary conditions. Mange, mastitis, diseases of the respiratory tract, mineral deficits, digestive problems, and parasites are among the most common [13, 14]. New and little known diseases of camels have been reported in recent years [6, 12, 15, 16], and this has been achieved on the basis of the knowledge and observations of local communities. Indeed, herders’ observations on camel illnesses are often a necessary step for veterinary scholars who are investigating aetiology, symptoms, and treatment of camel diseases [12, 15].

The study and application of this knowledge, and its integration in mixed veterinary systems, are important in all those contexts where pastoral populations have developed complex forms of observation, interpretation, and treatment of diseases affecting livestock [17]. In addition, veterinary remedies based on local knowledge are often more readily available, less expensive, and culturally more accepted than conventional treatments, especially in the case of nomadic populations living in arid and remote areas [1, 18]. With the ‘*knowledge of local knowledge*’ [1], scholars and international organizations pursuing development and cooperation projects can achieve a better understanding of existing ethnoveterinary practices (also by means of a shared vocabulary and definitions of diseases and their treatments), identifying headline priorities in terms of animal health from a local/emic perspective [1, 19].

In this paper, Sahrawi conceptualization of camel diseases and their treatment and remedies based on minerals, animals, and plants characteristic of desert habitats are explored and discussed.

**Fig. 1:**
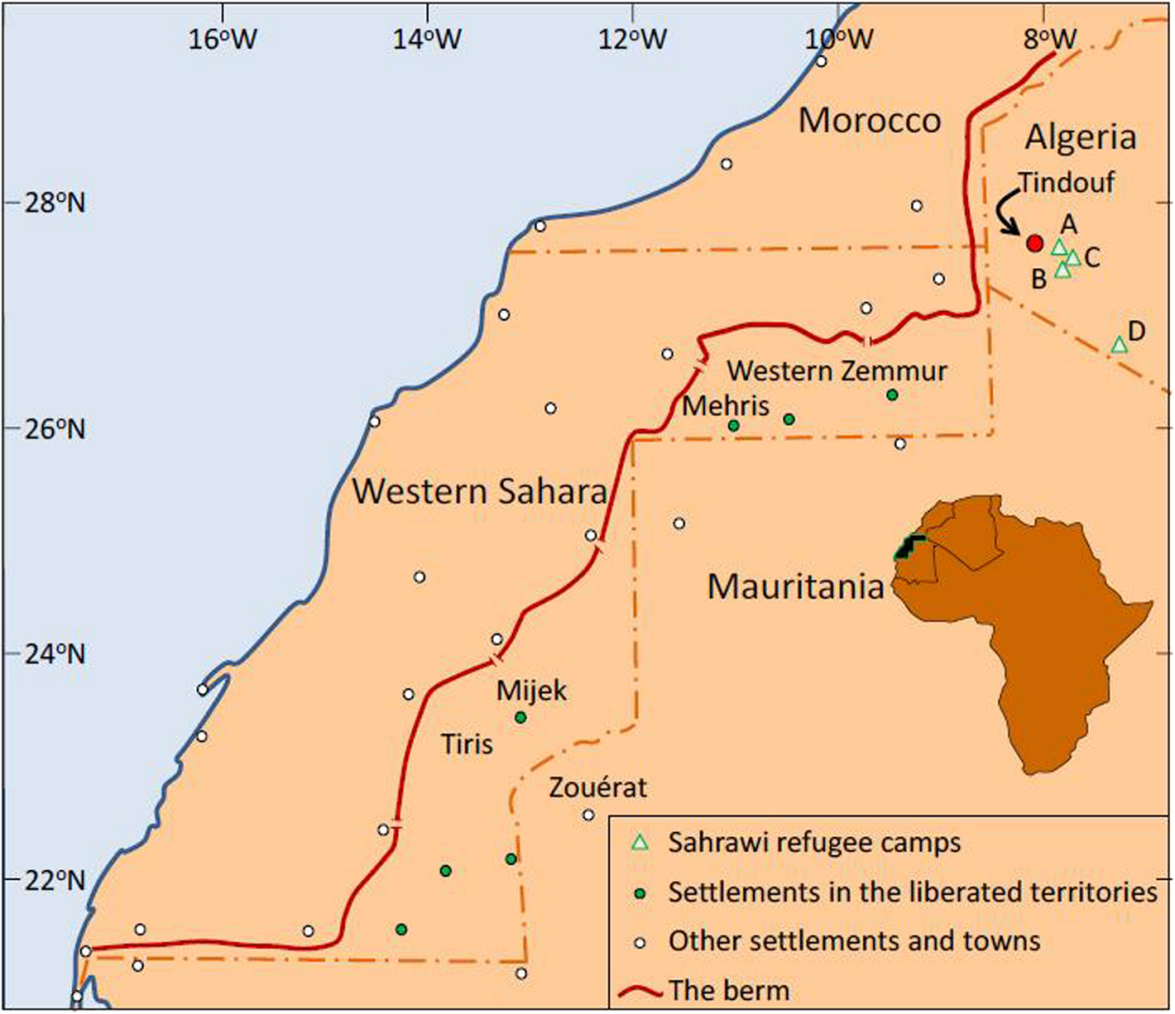
Map of Western Sahara and geographic extent of the study area

The Sahrawi, literally ‘people from the desert,’ are pastoralists who traditionally inhabited the coastal areas of Northwest Africa, including Western Sahara, northern Mauritania, and part of Southwest Algeria. The Sahrawi were a nomadic population, raising camels, goats, and sheep in the low-lying plains of Western Sahara and relying for food on livestock products as well as dates, sugar, cereals, and legumes bartered for livestock in markets surrounding their catchment areas [20]. In 1975, as a consequence of the Moroccan occupation of Western Sahara, about 70,000 Sahrawi were forced to flee and settle in refugee camps established in neighbouring Algeria [21], which led to a 16-year war ensued between Morocco and the Sahrawi’s armed political organization, the Polisario Front (1975–1991). About 165,000 Sahrawi live nowadays in four refugee camps located on the *Hamada* desert plateau within the Tindouf region of Algeria (Fig. 1). Food, shelter, and other basic commodities are provided by the European Union through bilateral development programmes, UN agencies, the Algerian government, and several solidarity groups [21]. Nevertheless, livestock husbandry is one of the refugees’ few endogenous activities developed and recovered without any consistent attention or funding from donors and international organisations. Over the years, Sahrawi refugees have attempted to improve the quality of life in the camps, developing an informal economy, marketing a variety of basic commodities as well as food, and creating overland trade routes to the south (Mali and Mauritania) and the north (Algeria and Spain) [22, 23]. Economic activities have greatly increased since a UN-sponsored ceasefire agreement was signed with Morocco in 1991, which resulted in the demobilisation of the Polisario troops back into the camps and their re-engagement in livestock husbandry, seasonal nomadism, and trade [24].

**Fig. 2:**
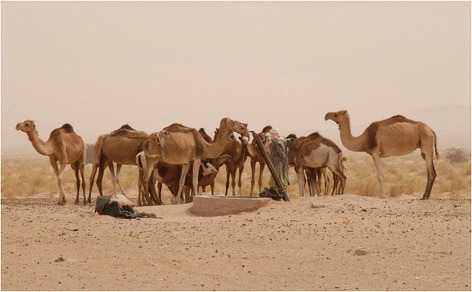
Sahrawi nomad with camel herd at a well (G. Volpato)

With the ceasefire agreement, the Polisario Front also assumed political control over the Eastern inland areas of the Western Sahara [25], the so-called ‘liberated territories’ (~20 % of the whole Western Sahara), while the remaining part is under the Moroccan Government’s administrative authority. Pastoral areas within the ‘liberated territories’ are important to the Sahrawi refugees’ struggle to maintain or recover traditional cultural and social practices, from livestock husbandry to medicinal plant use [26], as well as to generate income (e.g., through the sale of desert truffles) [27]. Some 20–30,000 Sahrawi who remained nomadic throughout the war by moving with their herds to safe areas in neighbouring Mauritania and Mali, also reoccupied these territories after 1991, herding back their livestock and using the refugee camps and Zouérat (Mauritania) as their main commercial hubs (Fig. 2). The revitalization of camel husbandry among Sahrawi refugees has been supported by the renewed interactions between refugees and nomads, which have led to a degree of fluidity between these categories. Both groups are using the ‘liberated territories’ (especially the northern areas) for grazing, and a dense solidarity and reciprocity driven network defines the structure of their social interactions [28]. Today, about 2000 camels are raised in the camps, whereas 40,000 head are present in the ‘liberated territories’ [29]. In the refugee camps, camel owners supplement natural forage with fodder purchased in Tindouf, whilst in the ‘liberated territories’ extensive nomadism (full-time and seasonal) is the most efficient strategy practiced for exploiting resources for herd production and reproduction.

The recovery of camel husbandry is ongoing in the refugee camps of the *Hamada* and in the ‘liberated territories’ where refugees travel and nomads live. The *Hamada* is a barren desert plateau known historically as the ‘Devil’s Garden’ because rainfall is scarce and edaphic conditions are poor, which explains why it has very poor forage resources, few trees, no halophytic plants, and few annuals. The climate of the ‘liberated territories’ is arid and continental with summer daytime temperatures above 50 °C, while during winter temperatures drop to zero at night. Rains generally occur from the end of the summer through autumn with an average annual rainfall of 30–50 mm; however, rains are highly irregular both within and between years, and droughts are recurrent. Despite this, the ‘liberated territories’ and especially the northern area (Zemmur) are richer in biodiversity and forage plants, displaying a savannah-like environment dominated by *Acacia* trees with flowering prairies found in flat gravel areas following rains [30]. The ‘liberated territories’ of Western Sahara are also those that provide Sahrawi refugees and nomads with mineral, animal, and vegetal materials at the basis of their ethnoveterinary practices.

Historically, camels were central to the production and reproduction of Sahrawi nomadic society, providing staple food, means of transport and warfare, commodity trading, and the social basis of power and prestige. Studies on Sahawi ethnoveterinary knowledge and their management of camels have been conducted by the first explorers and colonial scholars [31, 32]. Camels are still nowadays an important livelihood (e.g., provision of milk and meat, income) and cultural resource (e.g., for cultural identity) for Sahrawi nomads and refugees [24]. In order to manage their camel herds in arid lands, the Sahrawi have developed a deep ethnobotanical, ethnomycologycal, and ethnoecological knowledge [26]. Therefore, the Sahrawi have acquired a deep understanding and a complex conceptualization of the health status of their camels and, on the basis of this understanding (generated from trial and error, knowledge production, and transmission), they have elaborated practices and treatments to improve and restore camels’ health and productivity.

This study firstly presents the research methods applied during the investigation, followed by the results and discussion of Sahrawi knowledge of camel diseases and treatments, which are presented in three parts: 1) the cultural domain of camel diseases and their conceptualization; 2) treatments and remedies for camel diseases; 3) changes in knowledge and practices by contemporary Sahrawi. By addressing the little-known ethnoveterinary knowledge of the Sahrawi people, this study highlights the importance and level of details of the vast ethnoveterinary knowledge held by pastoralist populations.

## Methodology

The data analyzed in this study were collected in the Sahrawi refugee camps and in the Polisario-controlled ‘liberated territories’ of Western Sahara between 2005 and 2010. Fieldwork was carried out in accordance with standard guidelines for ethnobotany and ethnoveterinary investigation [33, 34], also including ethnobiological and anthropological methods such as freelisting, participant observation, and interviews [35, 36]. Freelisting exercises, used to describe the Sahrawi cultural domain of ‘*amradh al jemal*’ (i.e., camel disease) [37, 38], were conducted with 46 Sahrawi, both nomads and refugees, who were approached directly in their tents in either the refugee camps or in the ‘liberated territories’. Freelisting-generated data were used to stimulate qualitative discussion about the importance of specific illness and to study agreement among informants in cultural consensus analysis (CCA) [39, 40]. Details on the application of freelisting in cultural domain analysis using the very same sample of informants can be found in [28]. Follow-up semi-structured interviews were conducted with 36 informants (25 sampled from the group of the more knowledgeable informants extracted from the freelisting task and selected on the basis of their freelist lengths and experience with extensive camel nomadism; 11 further knowledgeable informants who did not participated to the freelisting task) to check unclear items in the lists, to investigate in greater detail the conceptualization of diseases (i.e., aetiology, epidemiology, symptoms, treatment, and prevention), and to collect data about treatment practices and remedies used. Interviews were conducted in *Hassaniya* (the Arabic language with a Berber substrate spoken by the Sahrawi), recorded and translated into Spanish by local research assistants. Informed consent was obtained verbally before interviews were conducted, providing an explanation of the aims, methodology, and outcomes of the study to participants. The ethical guidelines followed were those adopted by the American Anthropological Association [41] and by the International Society of Ethnobiology [42].

Regarding botanical methods, voucher specimens were collected with informants in the *Hamada* of Tindouf and across the ‘liberated territories’ through a ‘walk in the woods’ approach [43]. Plant nomenclature follows the Sahara and Western Sahara botanical standard treatises [44–47] and the International Plant Name Index (www.ipni.org). Voucher specimens were deposited in the National Herbarium of The Netherlands (Wageningen Branch–Herbarium Vadense).

## Results and discussion

### Camel diseases and disorders

The Sahrawi people hold vast camel-related knowledge, which includes knowledge about the environment, camel ethology and foraging behaviour, and camel health among others. The Sahrawi widely recognize strict and complex relationships between their camel herds and the ecosystem in terms of health and status. The factors regarded as the most important in maintaining a good health in camel herds are: 1) the potential for camels to move and wander and, consequently; 2) grazing and browsing freely from a variety of plants, including halophyte plants; 3) using the patched and seasonal resources of the desert environment. Thus according to Sahrawi pastoralists, camels mainly develop diseases as consequence of drought (i.e., when the patterns of movement and grazing are disrupted by lack of rains and grazing resources), sudden dietary changes and lack of movement (seen in camels raised in settled conditions and fed with hay, staw and leftovers), movement to areas where diseases or their vectors are present, and mineral (‘*salt*’) deficiencies. Table 1 presents the results obtained from the freelists on camel diseases of Sahrawi informants’ data using the CCA. Informants provided a cumulative list of 42 camel diseases, with individual freelists ranging from 0 to 25, and an average lenght per freelist of about 9.5 diseases. Among the diseases reported, some correspond to diseases recognized by Western veterinary medicine, whereas others show a correspondence with symptoms rather than with specific conditions and for others no references have been found in the Western veterinary medicine so far.

**Table 1:** The diseases cited are reported with their *Hassaniya* name in order of their frequency of mention, along with their western veterinary counterpart (when identified) or a short description of the disease, and the Smith’s index of salience

Disease	Diagnostic hypothesis	Frequency (%)	Smith’s salience index
*Jrab* ^a^	Sarcoptic mange	38 (82.6)	0.836
*Legrad* ^a^	Dermatomycosis	34 (73.9)	0.595
*Mhaz* ^a^	Respiratory infections	30 (65.2)	0.482
*Liren* ^a^	Mastitis	26 (56.5)	0.366
*Buguashish*	‘Kraff’ disease	23 (50)	0.399
*Rmah*	Crazyness (rabies, intoxications)	21 (45.6)	0.241
*Jidril*	Camelpox, contagious ecthyma	21 (45.6)	0.232
*Tukma*	Indigestion, alimentary diarrhoea	19 (41.3)	0.205
*Gargar*	Anthrax, clostridiosis	16 (34.8)	0.212
*Mindi*	Nutritional deficits, goitre	15 (32.6)	0.216
*Aulisis*	Salmonellosis	14 (30.4)	0.203
*Ghtaan*	Overwork syndrome	12 (26)	0.089
*Ghesh, homzi*	Internal parasites, colics	11 (23.9)	0.111
*Gushneda*	Abscesses, purulent lymphadenitis	9 (19.5)	0.075
*Sahrba*	Colostrum diarrhoea	9 (19.5)	0.053
*Zoran*	Salt deficits	9 (19.5)	0.119
*Bougueghir*	Edematous tumefaction, lymphadenitis	8 (17.4)	0.05
*Shedad*	Granuloma, organized abscess	8 (17.4)	0.06
*El Haibe*	Intestinal abscesses with pus infection	7 (15.2)	0.064
*Duda*	Caterpillar-borne abortion syndrome	7 (15.2)	0.053
*Larvar*	Cherato conjuntivitis, cornea opacity	6 (13)	0.068
*Tfadal*	Uterine prolapse	5 (10.8)	0.034
*Shdan*	Diffuse dermatomycosis	5 (10.8)	0.09
*Gushbeida*	Hypersalivation from ‘acid’ plants	4 (8.7)	0.041
*Dhbeb*	Trypanosomiasis	4 (8.7)	0.025
*El Helme*	Tick infestation	4 (8.7)	0.055
*Dhegbil*	Calves arthritis	4 (8.7)	0.025
*Sherghe*	‘Chocking’ syndrome	3 (6.5)	0.029
*Dharra*	Abscess under the toe	3 (6.5)	0.035
*Tinket*	Limp from trauma	3 (6.5)	0.027
*Maarguba*	Muscolar contraction, tendinitis	3 (6.5)	0.013
*Borues*	Head oedema/inflammation	3 (6.5)	0.02
*Tetrah*	Infected mastitis	3 (6.5)	0.035
*Tergan*	Indigestion from argan seeds	2 (4.3)	0.012
*Kirkle*	Chest-pad infections	2 (4.3)	0.01
*Burgheiba*	Wry neck syndrome	1 (2.2)	0.018
*Meshmusa*	Sunstroke	1 (2.2)	0.002
*Tabal*	Dystocia	1 (2.2)	0.004
*Telhad*	Tumoral degeneration of the hump	1 (2.2)	0.006
*Nthep*	Dislocation of rear legs’ articulations	1 (2.2)	0.001
*Legma*	Flea infestation	1 (2.2)	0.007
*Trakbin*	Knee inflammation	1 (2.2)	0.013

^a^ disease included in the cultural consensus model

Sarcoptic mange and dermatomycosis were reported respectively by 83 and 74 % of the informants, with mange having the highest overall salience index. Respiratory infections (e.g., pneumonia and common infections of the cold season) were the third most salient disease mentioned by 65 % of the informants, while mastitis (57 %) ranked fourth. These four were cited by more than half of the informants, forming the cultural consensus model of shared knowledge of the domain, as calculated by ANTHROPAC. This model represents domain items most likely to be mentioned by a typical member of the sampled population, due to the frequency and ranking of the reports provided by the informants. Ten diseases were mentioned by more than a third of the informants (e.g., *buguashish* or *kraff* disease, camelpox, digestive problems and clostridiosis), a pattern that both confirms the existence of the domain and demonstrates a core of shared knowledge about its contents.

Table 2 presents the results obtained from ANTHROPAC’s consensus analysis of the disease freelists. The first factor explained 77.7 % of the variation in informants’ performances (λ = 22.73), the second factor 18.1 % (λ = 5.29), whilst the third factor 4 % of the variation (λ = 1.23). Since the ratio of the first factor to the second is >3, this indicates high overall agreement among informants and, therefore, that they belong to a single culture (i.e., shared knowledge of camel diseases). The second factor suggests enough residual agreement to indicate subgroups, or clusters of agreement, within the study population [39]. It also indicates that knowledge on this cultural domain is undergoing change, likely due to the reduced importance of camels among the Sahrawi seen in the last 50 years, the relatively small number of refugees’ households who are engaged in camel husbandry at present, and the ongoing shift from strictly nomadic to intensive camel management.

**Table 2:** Consensus analysis results for Sahrawi freelists on camel disease expressed as eigenvalues decomposition

Factor	Value (%)	Ratio
1	22.731 (77.7)	4.292
2	5.296 (18.1)	4.283
3	1.237 (4.2)	
TOT	29.264 (100.0)	

Pseudo-Reliability = 0.976

Here below are discussed the camel diseases included in the cultural consensus model and few others of interest. A particular focus is given to *buguashish* and associated treatment/recovery practices for being a relatively unknown condition of complex conceptualization.

#### Mange and dermatomycosis

Sarcoptic mange (*jrab*) is the most reported camel disease, mentioned by more than 82 % of the informants, while dermatomycosis (*legrad*) is ranking as second and reported by three-quarters of the informants. Mange is a contagious skin disease characterised by crusty, pruritic dermatitis and hair loss, caused by mites of the family Sarcoptidae (*Sarcoptes scabei* var. *cameli*) [48]. The Sahrawi recognise that the clinical status of the affected individuals worsens with periods of drought, the cold season, and food deficits, conditions in which mange progresses quicker. Studies have shown that nutritional deficiencies, especially of Vitamin A, are conditions that predispose to mange [49], also considering how mange does tend to spread more quickly in cold weather when animals increase the contact rate huddling together [50]. Separation of herds and isolation of affected animals are practiced especially during the night.

Dermatomycosis is produced by a wide variety of soil-inhabiting yeasts and moulds of primary or secondary origin. It is a common pathology that affects mainly calves and young individuals, and which is well known and recognized by Sahrawi herders. About 10 % of the informants also mentioned *shdan*, described as a diffuse dermatomycosis where lesions are characterised by exudative secretions with scab formation.

The Sahrawi treat mange and dermatomycosis with several remedies, mainly vegetal tars and oils or fatty substances, all topically applied on the affected parts. Tars were historically obtained from the local vegetation [51, 52], for example by long cooking in water the aerial parts of *Hammada scoparia* and/or *Euphorbia granulata*, or the leaves and aerial parts of *Rhus tripartita*, *Acacia tortilis* and *Anabasis articulata*. Fat substances used to treat mange and dermatomycosis included the application of camel milk, fermented goat milk, milk cream and butter, camel hump, and of a mixture of butter or animal fat (e.g., from camels’ hump) mixed with grinded charcoal obtained from *Calotropis procera*’s stems. Remedies based on fats and congener plants used by the Sahrawi are also reported among the Tuareg of Niger [53] and camel pastoralists of the Qassim region of Saudi Arabia, where mange is treated with a plaster obtained from aerial parts of *Tamarix aphylla*, *Blepharis ciliaris*, *Hamada elegans* and *Euphorbia cuneata* triturated and mixed in melted butter [10].

#### Mhaz

*Mhaz*, mentioned by about 65 % of the informants, refers to infections of the respiratory system such as pneumonia, pleuropneumonia and/or pseudotuberculosis, as well as to respiratory syndromes of difficult interpretation and recent discovery [12]. The Sahrawi define *mhaz* as a respiratory condition affecting individuals of both sexes and of any age, often progressing to death [32], and describe symptoms such as ‘*the lungs of the camel get stuck to the ribs*’, dyspnoea, catarrhal inflammation with persistent cough, and occasionally bloody discharge. The affected camel tends to isolate itself and squat, which is considered to aggravate the condition. Individuals with *mhaz* are separated from the rest of the herd and forced to run, allegedly to increase their respiratory rate and thus facilitate mucus expulsion. The observation that camels recover by expelling big volumes of mucus has probably lead Sahrawi herders to develop treatments aiming to achieve this as a sign of recovery. Similar practices can be found among the Tuareg, who also force camels to run in order to promote nasal drainage and thus mucus expulsion [53, 54]. As an alternative treatment, cauterization is practiced on the nose and/or at its sides, at the anterior triangle of the neck under the mandible and/or on the throat, or on the chest. A further therapeutic technique includes the incision of the lower part of the nostrils so that the irritation forces the animal to sneeze and expectorate the mucus. Fumigations with green plants of *Hammada scoparia* and *Panicum turgidum*, among others, are also attempted. Nowadays Sahrawi herders use available antibiotics as a treatment for *mhaz*, especially in the refugee camps and with market-oriented husbandry.

*Mhaz* has many similarities with *sonbobe*, a camel respiratory disease described in Ethiopia and characterised by cough, nasal discharge (occasionally with blood) and inappetence [12]. Lung lesions and adherence of the lungs to the thoracic cavity were findings of the post-mortem investigation, as similarly reported by the Sahrawi in relation to *mhaz*. The use of oxytetracyclin significantly reduced the mortality [12, 16]. *Sonbobe* was identified as pneumonia caused by *Pasteurella haemolytica* (pasteurellosis), being climatic changes and physical stress in association with viral infections potential predisposing factors [12].

#### Mastitis

Mastitis has an economic and zoonotic importance in livestock husbandry, and in camels it makes no exception, as it is a main constraint that camel owners face in milk production [55]. Different pathological types of mastitis are known, and several bacteria have been identified to cause the infections [56]. Camel milk was a staple food among Sahrawi nomads and, since mastitis reduces and/or spoils milk production, it is considered an important camel health problem. The Sahrawi recognize a direct correlation between mastitis and herd hygienic conditions, reporting the main causes of mastitis as: 1) housing animals during the nights in small pens with accumulation of faeces, a common condition in the refugee camps, and 2) engorgement of the udder due to milk retention. Three informants described with the term *tetrah* a form of suppurative mastitis with presence of blood in milk. While mastitis due to milk retention is usually treated by frequent milking, mastitis of bacterial origin are treated with topical applications of plasters obtained by mixing oil or fat with the grinded aerial parts of *Zygophyllum gaetulum* (sometimes in combination with *Artemisia herba-alba*, *Asphodelus tenuifolius* and *Panicum turgidum*), boiled desert truffles (*Terfezia* and *Tirmania* spp.) [27], smashed and boiled aerial stems of *Cynomorium coccineum*, or the decoction of grinded seeds of the argan tree (*Argania spinosa*). A further traditional remedy consists of topical applications of the dried skin of spiny-tailed lizards or chameleons, which are believed to ‘*absorb*’ the infection. Medial and lateral cauterizations of the udder are also performed.

#### Buguashish

Half of the informants mentioned *buguashish*, a salt deficit that affects prevalently camels under 4 years of age and milking she-camels, particularly during drought or when they pasture or travel in sandy areas of Northeast Mauritania, Southwest Algeria, and Mali. *Buguashish* starts with a clear symptomatological feature: front limb lameness. This pathological condition is believed to be caused by pain resulted ‘*from the adherence of the lungs to the ribs*’, ‘*a pain to the chest*’. Indeed, the etymological root of *buguashis* comes from *deshush*, which means ‘chest’, and *buguashish* can be translated as ‘chest pain’ (or also from the word *lgashush*, i.e., the front legs [32]). The abnormal gait linked to a swollen and painful chest lead in few days to the animal’s inability to stand. Other symptoms include weakness and tiredness with tendency to fall, general apathy and loss of appetite, which are often associated with a ‘*dry cough*’ or a rather guttural noise (i.e., due to the breathing discomfort caused by broken ribs). A calf born from a she-camel affected by *buguashish* will usually display weakness to the limbs and poor health status. Sahrawi pastoralists associate *buguashish* to sandy areas and identify the etiological cause in a prolonged travel in dune-dominated areas, reporting how ‘*buguashish happens to camels that walk for too long in soft soils*’. However, most informants conceptualize *buguashish* in terms of presence or absence of important salty pastures, collectively called *hatba* by the Sahrawi; i.e.*, Nucularia perrinii*, *Salsola*, *Traganum*, and *Atriplex* species [28]. In sandy areas, the only species of *hatba* available for camels is *Cornulaca monacantha*, which has a rather patchy distribution. Given the importance and geographical distribution of *N. perrinii* in Western Sahara, Sahrawi herders tend to associate its presence with healthy camels and its absence (e.g., eastward from Zemmur and Tiris riverbeds and gravel plants) with mineral deficits and other diseases. Therapy relies in moving camels to areas where they can graze *Nucularia perrinii* (or ‘*to rocky soils*’ where this species grow), alone or in association with other salty plants. If movement to such areas is not possible, cauterization on the chest, washes with very cold water, and feeding of grounded bones, especially donkey bones, mixed with water are attempted. The latter treatment is based on herders’ observations that camels often practice spontaneous osteophagy, especially so when affected by *buguashish* (i.e., ‘*no matter what, if a pregnant camel find a bone will start licking it*’; ‘*when you see camels busy on carcasses, they are looking for the bones because they miss salt*’).

Camels are known to graze and browse from a variety of plants, with halophyte plants providing the primary source of salts and minerals. Mineral deficits and correlated conditions are relevant in many desert areas where camels usually live [57]: a NaCl deficiency can cause skin necrosis, whilst a chronic deficit can lead to neurological syndromes; serum phosphate/calcium disequilibrium leads camels to an illness called *kraff* (or *krafft*), as reported in North African countries [58]. *Kraff* has been investigated only in South Tunisia [59, 60], but it has also been observed in Southeast Algeria [61] and in very arid areas of North Africa, particularly in sandy soils and grazing areas with low concentrations of chemically-available calcium and phosphorus [62]. *Kraff* is characterized by disorders of bone metabolism, which include spontaneous fractures and paralysis [49, 63, 64]. In a recent epidemiological study [59], the disease has been described to primarily affect 2-year-old camels, year after year at the end of winter and in spring, especially among winter-transhumant herds. An analysis of the mineral composition of grazing plants present on the transhumance areas has shown that these plants are rich in calcium and poor in phosphorus (no one of the plants reported belonging to the Chenopodiaceae family), whilst a marked hypophosphatemia and hypercalcaemia were reported in affected animals [59]. The symptomatology includes abnormal gait, osteoarticular oedemas, spontaneous rib fractures, difficulty maintaining the posture with pain and tumefaction mostly localised on the knees, a clinical picture that further progress to anorexia, paralysis, and resulting in the death of the individuals in 1 to 4 weeks [49, 61]. In addition, conditions like rickets and osteomalacia are caused by a deficiency or impaired metabolism of calcium, phosphorus, or vitamin D. Osteomalacia occurs in mature camels and is characterized by general weakness and lameness, stiffness, emaciation and easy fracturing with permanent recumbency and eventual death occurring in the final stages. In Inner Mongolia, osteomalacia and its associated symptoms (e.g., fractures) among Bactrian camels have been related to low concentrations of phosphorus and copper in bones, kidneys, and liver, along with a high Ca:P ratio in forages present in sandy deserts [65]. Phosphorus deficiency is the most prevalent condition affecting grazing livestock throughout the world [66–68], especially in tropical areas, with increasing phosphorus availability only during the short periods of the early growth stages of plants. Phosphorus deficiency in grazing livestock has been also related to lameness, which in turn would further decrease movements and hence feed intake, pushing individuals to death from exhaustion [68]. Although relatively few studies on this topic have been conducted on camels, it is becoming clear that camels reared under extensive nomadic conditions are prone to phosphorus deficiency in those areas where soils are poor in this element.

The main features that these conditions have in common are their clinical manifestation in phosphorus-poor sandy areas and/or during drought, with their symptoms involving the osteo-articulatory system (e.g., lameness, joint enlargement, rib fractures, etc.). A further shared characteristic is the peculiar behaviour of affected camels consisting in licking and ingesting salt-rich substrates, such as bones, rocks, and soil. The soil and bones ingestion by camels with mineral deficiency is reported by several African pastoralist populations [49, 66], with osteophagy being attributed by Somalian pastoralists to a dietary phosphorus deficiency [69]. In the Algerian Beni-Abbes, camels have similarly been reported to feed from bones, ‘*especially on hot and sandy soils*’ [70]. These facts hint to a wide incidence of mineral deficiency in camels across arid and desert areas of Africa. Therefore, *buguashish* may represent a nutritional and mineral deficiency syndrome, especially occurring during drought and when camels graze for long periods in phosphorus-poor soils. Problems related to phosphorus deficits become evident in phosphorus- and halophyte-poor sandy soils, such as those at the eastern periphery of the Sahrawi nomadic territories where *N. perrinii* is not present. Thus, the conceptualization of *buguashish* in Sahrawi culture, in absence of knowledge of the phosphorus and its role, was built around a counterposition of positives (*N. perrinii* and other salty plants, healthy status, associated with rocky soils–identified in the gravel plains of Zemmur and Tiris) and negatives (no *N. perrinii*, fractures and lameness in camels, associated with sandy soils–identified in the dune areas at the eastern periphery of their customary nomadic territories).

Besides *buguashish*, the Sahrawi recognise other conditions related to nutritional deficiencies: ~20 % of the informants reported an illness related to generic salt deficits called *zoran*, whereas *mindi* (mentioned by one-third of the informants) is also associated with the absence of *N. perrinii*, and is characterised by swelling of the neck’s ventral part, anorexia, weight loss and death. Camels can recover ‘*with warm temperatures and salty plants*’. This description points to nutritional deficiency and primarily to goitre (i.e., iodine deficiency) as a diagnostic hypothesis for *mindi*. Goitre could be due to the lack of iodine in soils and grazing areas, and similar conditions of iodine-poor soils and forage plants causing nutritional deficiency to grazing camels have been reported in Darfur [71]. As described by Tuareg nomads of Agadez [11], goitre manifests in camels with a soft swelling of the ventral part of the neck, which can lead camels to death due to the potential compression of the trachea.

#### Rmah

More than 45 % of the informants reported *rmah* in the list of camel diseases they recognise. *Rmah* refers to ‘*crazyness*’ and includes onsets of rabies as well as of plant poisoning (e.g., from seeds of *Astragalus vogelii*, *Trigonella anguina*, or of *Battanderia amoena*, named respectively by the Sahrawi as *fenter*, *el gard*, and *aliat*) associated with neurological and behavioural symptoms: camels kick, lack of coordination, and ‘*eat and suddenly start running*’. Other symptoms include ‘*anarchic behaviour*’, ‘*staggering*’, and ‘*wandering as if they were blind*’ [31]. The same clinical condition has been reported by Tuareg of Niger with the name *atoufi* [53]. These symptoms are usually seen in camels and are caused by rabies, clostridiosis, intoxications, encephalitis, or cerebral congestion due to long exposures to direct sunlight without enough water intake [31], or sometimes also due to a massive infestation of *Cephalosporina titillator* larvae [53]. Cases of rabies in camels have been reported in Mauritania [72] and more recently in Niger [73], especially when sharing the same ecosystem with feral or domestic dogs, as it is also the case in and around the Sahrawi refugee camps.

#### Gargar

Almost 35 % of the informants mentioned *gargar*, also known as ‘*sudden death*’. The affected camel runs, jumps, bites its own hump, shows clinical symptoms such as dry cough and fever, always resulting in death [31]. Just before dying, the camel turns its neck backwards and produces a vocal noise described as ‘*gagaga*’, from which *gargar* may have an onomatopoeic origin [32]. *Gargar* has an acute or sub-acute form, with death of affected individuals occurring minutes to few hours after the initial onset. Healthy adult animals are prevalently affected before grazing in the early morning hours, or returning from grazing on green pastures. Its sudden onset without known prevention or treatment is often explained by herders in fatalistic terms, such as ‘*disease of the devil*’ [31]. Sometimes diarrhoea is also reported but on its interpretation Sahrawi herders do not agree; the majority regards it as a sign of the fatal outcome, some others as a possible sign of recovery helping to clear up the illness. The etiological causes of *gargar* are unknown but the disease is associated with rains (and the consequent availability of green pastures) and with specific areas, especially sandy and dune areas [31]. The potential transmission route of *gargar* by eating carcass’s bones of affected animals is stressed by Sahrawi informant as specific of the disease. Many informants state that *gargar* can be transmitted from camels to man (through ‘*smelling*’ a camel that died of *gargar*), manifesting with carbuncles and abscesses which can lead to a chronic weakness condition or even death. In the annals of the Reguibat L’Gouacem [20], the year 1915 is known as ‘*the year of the dait el gerger*’, in reference to a rain pond causing sudden camel deaths. The Tuareg of Niger also report a disease called *tandar* (a term which means ‘*brutal death*’) characterized by rapid putrefaction and bleeding from body orifices. This disease has been identified as anthrax [11], whilst different studies also attribute anthrax to *gargar* [31]. Dormant endospores of *Bacillus anthracis* can survive in extreme conditions for decades or even centuries, reactivating and multiplying rapidly when inhaled or ingested by grazing herbivores. Others (e.g., carnivores, humans, etc.) become infected by consuming or coming into contact with infected carcasses.

#### Homzi

*Homzi* (or *ghesh*) was reported by ~24 % of the informants and is related to camels’ ingestion of dew. Sahrawi herders report *homzi* as typical of South Morocco, where plants grazed are ‘*wet*’ or ‘*humid*’, therefore linking this as causing *homzi*. Camels with *homzi* are weak, with a swollen throat, dry cough, diarrhoea, and present colic. As a preventive measure the Sahrawi do not allow camels to graze during the first hours of the morning, when plants are still full of water droplets resulting from the night condensation. This condition is also present in the annals of the Filala and Izarguien tribes [20]: the years 1931 among both tribes and 1940 for the Filala only are known as ‘*the year of gosh*’, an illness described as been caused ‘*by feeding from pastures with dew, which causes an abscess in the throat*’. A similar clinical condition has been reported among the Tuareg of Niger [53] and among Somali herders [74].

Camels can be infested by several endoparasites (e.g., *Strongyloides*, *Trichuris*, *Tricocephalus*, *Haemoncus longistipes*, etc.), with acute helminthiasis being often associated with diarrhoea and weakness [50, 75]. The main diagnostic hypothesis for *homzi* is a parasitic disease caused by strongyles, whose larvae migrate at the top of the grasses during the coolest hours of the day (i.e., in the early morning), ready to be ‘*grazed*’ [76]. This may explain the prevention system developed by Sahrawi and Saudi Arabian herders [13]. A well-established and known correlation between strongyle infestations and dew exists [18] and, although the Sahrawi have no knowledge of the strongyloid’s existence and life cycle, nevertheless they elaborated successful strategies based on their observations and learnt to move to drier areas as soon as *homzi* symptoms appear.

#### Trypanosomiasis

Although trypanosomiasis is one of the diseases most dreaded by camel pastoralists and the camel’s southern distribution in Africa is defined by the presence of the main trypanosomiasis vector (the tsetse fly–genus *Glossina*), trypanosomiasis was mentioned by just four Sahrawi informants (~9 % of the total interviewed).

Trypanosomiasis caused by *T. evansi* (so-called *surra* or *dhbeb*–lit. ‘*fly*’ among Arabic-speaking populations) is the most important single cause of morbidity and mortality in camels in many African countries (e.g., Kenya, Somalia, Niger) [77, 78], and is generally present during rainy seasons in most of the remaining camel husbandry areas including Morocco, Central and Southern Mauritania, Mali, Chad, and Niger [79–81]. In north-western Africa, trypanosomiasis is reported having a low prevalence in grazing areas of Tafilalet and Ouarzazate provinces, Morocco [81]. It appears that seroprevalence progressively decreases with decreasing in annual precipitations towards the inner Sahara desert, whilst it is probably negligible in drier areas of Western and Central Sahara, at least according to local pastoralists’ knowledge. Unpublished data from studies conducted in the area did not revealed clinical disease or parasitemia by analysing blood smears and buffy coats (Broglia, personal communication). Zemmur and Tiris, the two main nomadic areas of Western Sahara, are historically reported as free from trypanosomiasis within the Western Africa region [31, 32], mainly due to the lack of palm oasis and permanent water surfaces that would enable vectors maintenance and reproduction [82]. The Sahrawi knowledge about trypanosomiasis is rather related to those areas surrounding their traditional nomadic routes (e.g., southern Mauritania and Mali to the south; south Morocco to the north), which were used for commercial exchange (i.e., caravans) and as emergency grazing areas. In fact, this disease is known and recalled only by those Sahrawi nomads who moved their herds into endemic areas. According to informants, the animal affected by *dhbeb* progressively ‘*loses weight, strength, sometimes becomes blind and eventually die*’. Death can be delayed or health restored if affected camels are moved to inland Western Sahara to graze from *Nucularia perrinii*, which is here used as exemplificative of the trypanosomiasis-free Western Sahara environment (i.e., if camels graze from *N. perrinii* it means that they are in Western Sahara and thus not exposed to trypanosomiasis vectors). Like for *buguashish* and *mindi*, the Sahrawi’s understanding of trypanosomiasis is embodied in the contrast between their customary areas of Western Sahara (where the disease is absent and *N. perrinii* is abundant) and areas further to either the South or the Northwest Sahara (where the disease is present and *N. perrinii* is absent).

### Ethnoveterinary remedies

In accordance to how each disease is understood and culturally constructed, the Sahrawi, in their long-term engagement with camel husbandry and the desert environment, have developed an array of veterinary practices with treatments largely based on desert plants, animals, and minerals.

#### Vegetal remedies

**Fig. 3:**
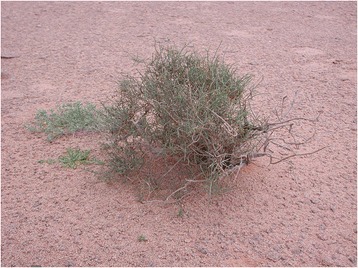
*Hammada scoparia* in Zemmur (G. Volpato)

The use of plants or parts of plants in traditional ethnoveterinary systems is widespread worldwide among pastoral and agro-pastoral populations. In Table 3 are listed 42 plant species from 22 botanical families used by the Sahrawi for treating camel diseases and veterinary conditions, in order of their scientific name, along with the local (*Hassaniya*) name, voucher specimen number, parts used (and their local names, where applicable), preparation and administration route. The most represented botanical families are Chenopodiaceae (6 species), and Euphorbiaceae, Fabaceae, and Poaceae (4 species each). The most cited species in terms of number of individual reports and of different veterinary uses are *Hammada scoparia*, *Acacia tortilis*, *Zygophyllum gaetulum*, *Nucularia perrinii*, and *Panicum turgidum. Hammada scoparia* is a resistant plant that grows in arid environments of the Mediterranean basin, North Africa, and the Middle East. In Western Sahara, it is very abundant in Zemmur (Fig. 3), while it progressively disappears moving southward to Tiris. It is known by the Sahrawi, as well as most Arabic speaking countries, as *rimth* or *remth*, and is widely used in the ethnoveterinary medicine in the countries where it grows [13]. In almost 50 % of the therapeutic applications, leaves and aerial parts are used, followed by seeds (15 %), bark (12 %), and fruits (8 %). In 5 % of the cases plant latex is used (e.g., from *Calotropis procera* and *Euphorbia* spp.) mostly through topical applications in the treatment of mange and wounds. The administration routes of remedies, based on ointments, plasters, or extracted juice preparations, is through topical application in 55 % of the cases; in 23 % of the cases by oral route (i.e., mixed with food or suspended in water); drenching and dip-baths using decoctions or infusions of plants in 5 % of the cases each. Therapeutic fumigations with green plants have been reported for 12 % of the ethnoveterinary treatments. Fumigations, with the animal ‘*wrapped in smoke*’, are used in the treatment of camelpox and contagious echtyma, *mhaz* and other respiratory infections, *buguashish*, *aulisis*, and tick and flea infestations. Their specific purposes include prevention of secondary infections (e.g., at the sites of skin ulcers in camelpox), disinfection, and as arthropods’ repellent (e.g., of ticks). Green plants used include *Nucularia perrinii*, *Salsola* spp., *Panicum turgidum*, *Launaea* species, and dry *Euphorbia echinus*, with the aerial parts of *Hammada scoparia* and *Zygophyllum gaetulum* as the first choice. Plants are likely to be selected on the basis of their availability in the desert environment (i.e., plants used for fumigations are among the most common evergreen plants in Western Sahara desert) and for their established use in traditional medicine. The use of plant fumigations for medicinal/repellent purposes is well established among pastoralists worldwide, where peculiar is the use of mugwort’s (*Artemisia vulgaris*) fumigations among nomads of Inner Mongolia to protect livestock from yellow mosquitos swarms [83].

**Table 3:** Plant used in Sahrawi camel ethnoveterinary practices

Botanical taxon	Family	Voucher specimen	Local Hassaniya phytonym	Parts used (Hassaniya name)	Preparation and use	Indications
*Acacia ehrenbergiana* Hayne	Fabaceae	GV1015	*Tamat*	Fr (*jarrub*)	Mixed with fodder	Dietary supplement (nutraceutical)
		GV1058				
*Acacia senegal* (L.) Willd. var. *senegal*	Fabaceae	GV1076	*Amour*	Fr (*sallaha*)	Dried, grinded and given as powder (in winter time), or mixed with water (during hot period)	Diarrhoea
				Ba		Indigestion
*Acacia tortilis* (Forssk.) Hayne subsp. *raddiana* (Savi) Brenan var. *raddiana*	Fabaceae	GV1010	*Talha*	Le (*warga talha*)	Mixed with fodder	Dietary supplement (fattening)
				Fl (*anish*)		
				Fr (*jarrub*)		
				Ba	Grinded (topical application)	Wounds cicatriser
				Re (*al elk talha*)		
		GV1025				
				Le (*warga talha*)	Grinded and mixed with sugar and oil	Diarrhoea
				Le	Leaves are burnt and a decoction is made; a plaster is prepared with water and coal obtained from the bark of *talha* and *shdari* (topical application)	Sarcoptic mange
				Ba		
*Allium cepa* L.	Alliaceae		*Besla*	Bu	Heated in oil, given as food supplement once per day during 2 or 3 days or until the animal gets better (topical application and vaginal washes)	Cough
						Post-partum prolapse
						Abortion
						Camelpox
						Mastitis
*Allium sativum* L.	Alliaceae		*Thoum*	Bu	Heated in oil or fried (topical application)	Post-partum prolapse
						Mastitis
*Ammodaucus leucotrichus* Coss. et Dur.	Apiaceae	GV1013	Kamuna	Ap	Heated in oil (topical application)	Skin ulcers
						Infected wounds
		GV1033	Kamunat rag			Mastitis
*Anabasis articulata* (Forssk.) Moq.	Chenopodiaceae		Ashram	Ap	Long-cooking in water with *warga talha* and *shdari* (topical application)	Sarcoptic mange
*Argania spinosa* (L.) Skeels	Sapotaceae		Argan	Se (*bulez*)	Inner part is grinded and then fried (topical application with *shmed*); argan oil can be used in the same way	Mastitis
*Artemisia herba-alba* Asso	Asteraceae	GV1042	Shih	Ap	Heated with barley peels and/or *aggaya* (topical application)	Mastitis
*Asphodelus tenuifolius* Cav.	Asphodelaceae	GV1078	Tazia	Ap	Heated with barley peels (topical application)	Mastitis
		GV2064				
*Atriplex halimus* L.	Chenopodiaceae	GV1052	Legtaf	Ap	Dried and grinded	It is a plant of *hatba* that can substitute *askaf*
		GV2061				
*Calotropis procera* (Ait.) Ait. f. subsp. *procera*	Asclepiadaceae		Tursha	St	The stem is burnt and ash is applied topically	Wounds caused by excessive or prolonged backloading
				La		
						Mange
				Ap	Dried aerial parts	Dietary supplement (nutraceutical)
*Camellia sinensis* (L.) Kuntze	Theaceae		Tcha	Le	Dried or heated in water without sugar	Diarrhoea
*Chamomilla pubescens* (Desf.) Alavi	Asteraceae	GV1090	Lerbien	Ap	Heated in oil (topical application)	Ulcers
						Infected wounds
*Cistanche phelypaea* (L.) Cout.	Orobancaceae		Dhenoun	Ap	Grinded and mixed with tar (topical application)	Mange
*Citrullus colocynthis* (L.) Schrad.	Cucurbitaceae	GV2068	Hadgit lehmar	Se	Cooked in water (plaster topically applied)	Mange
			Aferziz			
*Cleome africana* Botsch.	Capparidaceae	GV1026	Lemkheinza	Ap	Dried (when eaten in huge quantities it causes nervous disorders with tremors, ‘*as if it was drunk*’)	Dietary supplement (nutraceutical)
						Mange
					Green, grinded, mixed with *ludek* and topically applied to broken legs for 40 days; grinded, mixed with *ludek* and *nile* and topically applied to open wounds	Leg fractures
		GV2056	Mkheinza			Wounds caused by excessive backload
					Green, grinded and boiled in water during (water/plaster from the decoction topically applied)	Mange
*Cymbopogon schoenanthus* (L.) Spreng.	Poaceae		Idkhir	Ap	Grinded, mixed with water and topically applied as cataplasm; ash obtained from the aerial parts is applied topically	Wounds cicatriser
			Liedkhir			
*Cynomorium coccineum* L.	Balanophoraceae		Terzuz	Wp	Squeezed and the juice is given to calves; washes with the decoction water	Calves diarrhoea
						Duda syndrome
						Mastitis
*Euphorbia balsamifera* Aiton	Euphorbiaceae		Fernan	Ap	Fumigations are made with the decoction; latex (topical application)	Mange
				La		Tick infestations
*Euphorbia granulata* Forssk.	Euphorbiaceae	GV1055	Kbidet ed-dab	Ap	Decoction in water (plaster topically applied); grinded and topically applied after cauterizations	Mange
						Snakebites and scorpion stings
*Euphorbia officinarum* L. subsp. *Echinus*	Euphorbiaceae	GV1001	Daghmus	La	Added to drinking water; applied topically (latex can harm eyes)	Intestinal parasites
						Mange
*Hammada scoparia* (Pomel) Iljin.	Chenopodiaceae	GV1009	Remth	Ap	Squeezed and the juice is applied topically; boiled and used for washes	Snakebites and scorpion stings
		GV1021		Ap	Branches are burnt in front of the camel from one to three times, or once per week during some months	Camelpox
						Broncopneumonia
						*Oushri*
		GV1057				*Mhaz*
						*Buguashish*
						*Aulisis*
				Ap	Cooked (plaster topically applied); grinded and mixed with *alquitran*	Mange
				Ap	Dried and grinded (dietary supplement)	*Aulisis*
*Heliotropium ramosissimum* (Lehm.) DC.	Boraginaceae		Lehbaliya	Ap	Dried, grinded and mixed with water (plaster topically applied)	Ringworm
				Ap	Grinded (topical application with *nile* and sometimes salt)	Chest infection
*Hordeum vulgare* L.	Poaceae	GV2023	Zraa	Se (*leglya*)	Roasted seeds also mixed with water	*Ghtaan* (excessive workload)
*Lavandula* sp.	Lamiaceae		Lejzema	Ap		Mastitis
*Maerua crassifolia* Forssk.	Capparidaceae	GV1007	Atil	Ba	Burnt and grinded (applied topically inside the wound)	Wounds cicatriser
		GV1019		Le (*sadra el hadra*)		
*Mesembryanthemum cryptanthum* Hook. f. in Hook.	Aizoaceae		Afzu	Ap	Fresh aerial parts are grinded (topically applied)	Mange
*Nucularia perrinii* Batt.	Chenopodiaceae	GV1047	Askaf	Ap	Grazed	Dietary supplement
						Intestinal parasites
						Buguashish
						If *askaf* is the only
						pasture during drought periods, then mange develops faster
		GV2042				
				Ap	Aerial parts are burnt (inhalation)	Skin ulcers from camelpox
*Panicum turgidum* Forssk.	Poaceae	GV1051	Mrokba	Ap	Green plants are burnt (inhalation)	*Mhaz*
						Camelpox (maintain the infection at a low level)
			Umm rekba	Ap	Heated with barley seeds peels (topically applied); decoction with *aggaya* (topically applied)	Mastitis
*Peganum harmala* L.	Zygophyllaceae	GV1066	Harmal	Se	Heated in oil (topical application)	Strokes
						Abscesses
*Pergularia tomentosa* L.	Asclepiadaceae		Ghalqa	Le	Boiled in water; fumigations	Mange
			Umm lbena			
			Umm el-jlud	La		Ticks infestations
*Phoenix dactylifera* L.	Palmae		Tamra	Fr	Plaster made with the dates (topically applied)	Nails wounds
*Rhus tripartita* (Ucria) Grande	Anacardiaceae	GV1023	Shdari	Le	Decoction made with burnt leaves (topical application)	Mange
		GV1064				
		GV2021				
				Ba	Grinded (topical application)	Wounds caused by excessive backload
*Ricinus communis* L.	Euphorbiaceae		Aureuar	Se	Oil (topical application); seeds are grinded and mixed with milk/milk cream/animal fat (plaster topically applied)	Strokes
						Mastitis
						Udder inflammation
*Salsola imbricata* Forssk.	Chenopodiaceae	GV1054	Ghassel	Ap	Grazed	Intestinal parasites (regarded as acid plant able to treat intestinal parasites the diarrhoea caused by the plant)
*Salsola tetrandra* Forssk.	Chenopodiaceae	GV2020	Laarad	Ap	Grazed	Intestinal parasites (regarded as acid plant able to treat intestinal parasites the diarrhoea caused by the plant)
				Ap	Stems are burnt (inhalation)	Skin ulcers from camelpox
				Ap	Grinded (plaster mixed with *algatran* and topically applied)	Mange
*Tamarix* sp.	Tamaricaceae	GV1003	Ar’ar	Ba		Wounds caused by excessive backload
		GV1059		Wo		
*Terfezia ovalispora* Pat.	Terfeziaceae	GV1008	Terfes	Truffle	Filtered decoction applied topically; boiled ½-1 h and then grinded (plaster mixed with and topically applied once per day during 3 days or until the infection resolve); boiled and used for washes	Mange
						Mastitis
						Udder infections (*tedrenfut*)
*Trigonella foenum-graecum* L.	Fabaceae	GV1018	Halba	Se	A small quantity of seeds (as many as you can take between two fingers) are boiled in hot water (or the boiling pot of the second tea) then the liquid is drenched to camels two or three times; sometimes seeds are mixed with drinking water (acid plant that can cause abortion, not used during pregnancy)	Dietary supplement (nutraceutical)
						Diarrhoea or indigestion
		GV1044				Colic
*Triticum* spp.	Poaceae		Shir	Se	Milled and cooked until a thick plaster is obtained; mixed with drinking water	Indigestion
						Dietary supplement (during the hot season)
*Zygophyllum gaetulum* Emberger et Maire	Zygophyllaceae	GV1050	Aggaya	Ap	Boiled in water; fumigations are made with the cooled decoction	Mange
				Ap	Boiled and the resulting water is applied topically with a cloth once per day in the morning until resolved; heated with barley peels	Mastitis
		GV1065	El barraya			
				Ap	Milled or grinded and given to eat to the camel for three times	*Aulisish*

Part(s) used defined as: *Ap* aerial part, *Ba* bark, *Bu* bulb, *Ep* fruit epicarp, *Fl* flowers, *Fr* fruits, *Ft* flowering tops, *La* latex, *Le* leaves, *Ls* leaf stalks, *Re* resin, *Rh* rhizome, *Ro* root, *Se* seeds, *Sg* stigma, *Sh* shoots, *St* stems, *Tu* tuber, *Uf* unripe fruits, *Wh* young whorls, *Wo* wood, *Wp* whole plant

As reported in Table 4, vegetal remedies are used prevalently in the treatment of mange, wounds, and mastitis, explaining why topical applications are the most frequent administration routes. Comparing the number of herbal remedies used for each disease with the cultural consensus analysis, a correspondence for mange and mastitis can be clearly seen in that the most diverse remedies are used in the treatment of the most relevant diseases.

**Table 4:** Use of vegetal remedies for different camel diseases among the Sahrawi

Camel disease/condition	Frequency (%)
Mange	16 (19.8)
Wounds, skin ulcers and infections	12 (14.8)
Mastitis	12 (14.8)
Food supplement strengthener, nutraceutical	7 (8.6)
Diarrhoea, colic, digestive problems	7 (8.6)
Camelpox	5 (6.1)
Respiratory problems	4 (5.0)
Ticks, fleas, snakebites and scorpion stings	4 (5.0)
Intestinal parasites	3 (3.7)
*Buguashish*	3 (3.7)
Other skin parasitosis and dermatomycosis (e.g., tinea)	2 (2.5)
Reproductive problems (e.g., prolapse, abortion)	2 (2.5)
*Aulisis*	2 (2.5)
*Ghtaan* (overwork syndrome)	1 (1.2)
*Duda* syndrome	1 (1.2)
TOT	81 (100.0)

#### Other remedies

Other products than plants used by the Sahrawi in camel ethnoveterinary medicine (including processed products of plant origin) are reported in Table 5, along with their preparation technique and therapeutic use. Thirty remedies of animal, mineral, or other origins (e.g., industrial products) are listed. Although these categories of remedies are not as important as plant-based remedies, they are widely used for medical and veterinary applications around the world [84, 85], especially in arid ecosystems where vegetal resources are often temporally and spatially patchy. Among the Sahrawi, animals used as source of veterinary therapeutic agents include camels, goats, donkeys, spiny-tailed lizards, chameleons, and hyaenas. Camels are by far the main source of veterinary remedies: milk and milk cream are used to treat skin diseases such as mange; camel blood is put in eye drops to treat inflammations; cooked camel skin to treat gingivitis; the placenta is drenched to calves with diarrhoea. Besides traditional products, also ‘new’ remedies (e.g., based on products made available with modernisation and globalization) have been reported, such as bitumen and exhaust engine oil to treat mange, and insecticides against tick and flea infestations. Sources of minerals from bones and salt are used to treat nutritional deficiencies, whereas red ochre (*hemera*) is grated in water and drenched for internal strokes and fractures, or suspended in oil and topically applied on abscesses. Like for vegetal remedies, a majority of the remedies reported in Table 5 are used to treat skin conditions and wounds: several lipid-rich products (e.g., camel fat, milk cream and butter, oil, soap) are used to treat mange, dermatomycosis, and skin necrosis, whilst fats extracted from milk or camel hump are applied to open wounds (e.g., to the chest).

**Table 5:** Other products used in the treatment of camels in Sahrawi veterinary

Product	Preparation	Administration route	Indications
Bitumen	Mixed	Topical	Mange
Butter (camel/goat milk)		Topical	Chest wounds, Dermatomycosis
Camel blood	Mixed with sugar	Eye drop	Eyes inflammation
			*Larvar*
Camel fat (hump)	Mixed	Topical	Chest wounds Dermatomycosis
Camel placenta		Oral	Calf diarrhoea (preventive treatment)
			Duda syndrome
Camel skin	Coat is removed, skin is cut in small pieces and dried, then pit cooked in sandy soils	Topical	Gengivitis
Camel urine	Collected and plastered with the soil	Topical	*Shmel* (mastitis)
Chameleon skin (*Chamaeleo* spp.)	Dry	Topical	Udder infections
			Mastitis
Camphor		Topical	Mange
Donkey bones (*Equus asinus* L.)	Burnt, grinded and dissolved in water	Oral	*Buguashish*
Donkey faeces (*Equus asinus* L.)	Grinded and dissolved in water	Oral	Diarrhoea
Exhaust engine oil	Coat is cut	Topical	Mange
			Tick infestations
Fermented milk (camel/goat)		Topical	Skin conditions
Food crust	Collected from the bottom of the pot after cooking and then grinded	Topical	Wounds
Hematite/Red ochre [Iron(III) oxide Fe_2_O_3_]	Grinded and dissolved in water or oil	Topical	Hematomas
			Headstroke
			Broken ribs
			Abscesses
Hyena feces and/or blood (*Hyaena hyaena* L.)		Oral	*Aulisis*
Insecticide spray		Topical	Ticks
			Fleas
Milk cream (camel/goat milk)	Coat is cut	Topical	Mange
			Dermatomycosis
		Eye drop	Skin necrosis (*shdan*)
			Eye inflammation
			*Larvar*
Rock salt	Grinded and heated in fat	Topical	Camelpox
	Grinded and dissolved in water		Wounds
			Dietary supplement
Soap	Dissolved in water and oil, the floating fat is then collected	Topical	Mange
			Fleas
Spiny-tailed lizard (*Uromastix acanthinura* Bell.)	Roasted on the fire and cut in pieces, then dissolved in water	Oral	Kidney infections
			Abscesses
			*Aulisis*
			Weight and appetite loss
	Roasted, triturated and dissolved in oil	Oral	Duda syndrome
	Dried skin	Topical	Mastitis
Sugar	Dissolved in water	Inhalation	Diarrhoea
			*Buguashish*
Sulphur stone	Grinded and dissolved in hump fat, then left resting for a week	Topical	Mange
Tobacco	Heated in oil	Inhalation	Flies infestations
	Boiled or macerated in water	Dipping	Fleas
	Macerated in water	Oral	Duda syndrome
		Eye drops	
Vegetal oil	Heated	Topical	Dermatomycosis

#### Cauterizations

Besides treatments of vegetal, animal or mineral origins, the Sahrawi also made therapeutic use of cauterizations for about 21 diseases and/or health conditions such as mastitis, *mhaz*, *buguashish*, wry-neck syndrome, diarrhoea, abscesses and inflammations, and traumatic joint conditions. Cauterizations are performed with iron tools, knives, or hot embers, distinguished according to the type of technique applied. Cauterization is a therapeutic technique with ancient roots, being practiced in Europe and Asia using organic material more than 5000 years ago [86]. Nowadays, it is used in both medicine and veterinary medicine in different parts of the world [87], including the Sahara region [53, 88], where the present use of cauterization by Sahrawi pastoralists has been likely drawn from Arabic traditional practices. The Sahrawi distinguish three types of cauterization performed with iron sharp-edged tools heated over fire [32]: 1) linear cauterizations (*mahuar*), in parallel lines or cross-shaped patterns; 2) punctiform cauterizations (*keye*); and 3) penetrating cauterization (*nifde*). *Mahuar* and *keye* are used mainly to stimulate a reaction in life-threatening conditions (e.g., *buguashish*, calf diarrhoea, *mhaz*), for articular problems (e.g., arthritis, wry-neck syndrome), traumas, and swelling (e.g., of the head). These techniques are also used in the treatment of abscesses and purulent lymphadenitis (*gushneda*), of which the Sahrawi distinguish different types in accordance to their localization, etiology, and symptomatology (e.g., on the neck, at the basis of the tail, or of the hoof). Cauterizations are also used to treat *bougueghir* (mentioned by 17.4 % of the informants), which manifests with submandibular oedemas and is attributed to a long-time grazing on dried and stiff graminaceous plants (e.g., *Stipagrostis* spp.), whose barbels block the excretory duct of the salivary glands. In the penetrating cauterization technique (*nifde*), a fold of skin is taken between two fingers and pierced with an iron sharp-edged tool heated over fire. A thread, often coloured, is inserted as simple interrupted stitch. The thread is believed to ‘*draw out the illness from the blood*’, in the same way as the Masaai and Fulβe explain bloodletting [89].

From a western therapeutic perspective, cauterizations are considered to be effective in cases of abscesses and articulatory problems, but are likely to be of limited use in mastitis, kidney problems, pneumonia, chronic gastroenteritis and calf diarrhoea, as well as for general conditions caused by viral or bacterial infections. Nevertheless, they may play a role in stimulating the local circulatory system and thus possibly as a sterilisation technique, as well as in inducing a local hematogenic and immunogenic stimulation [90]. Sahrawi herders generally recognise that cauterization techniques may not be effective and often resort to them when no other remedy or treatment is possible or as a treatment attempt when they lack of detailed knowledge of the disease.

#### Mixtures

Sahrawi herders often use different products in combination to treat their camels. Table 6 reports the mixtures of plants and other products used. Four out of eleven mixtures are used to treat mange, three to treat mastitis and wounds, and only one for tick infestations. These mixtures are mainly combinations of remedies used in single recipes: plants such as *Ammodaucus leucotrichus*, *Artemisia herba-alba*, and *Zygophyllum gaetulum* are combined to treat mastitis; tannin-rich barks of *Acacia* species, *Maerua crassifolia* and *Rhus tripartita* are combined as wound cicatrizers; several other plants and products are combined in the treatment of mange. In some cases, the Sahrawi experiment in the use of remedies according to different concentrations, timing, and combinations, in order to achieve the best therapeutic effect.

**Table 6:** Mixtures used in Sahrawi ethnoveterinary medicine along with their lists of components (including the parts of plants used), preparation and indications

Plants/Other ingredients	Preparation	Administration route	Indications
*Ammodaucus leucotrichus* (se)	Plants are grinded and mixed with wheat flour, fried in oil and applied as a plaster with a cleaned cloth for 15 days, changing the plaster every day	Topical	Mastitis
*Lavandula* sp. (ap)			
Wheat flour			
*Allium cepa* (bu)	Fried, dissolved in a saline solution, then obtaining a plaster	Topical	Mastitis
*Allium sativum* (bu)			
*Acacia senegal* (ba)	Grinded and applied as cicatrizer for 5 days	Topical	Wounds
*Acacia tortilis* (ba)			
*Maerua crassifolia* (ba)			
*Euphorbia balsamifera* (ap)	Boiled in salt water, three washes per week	Dipping	Tick infestations
*Pergularia tomentosa* (ap)			
*Ammodaucus leucotrichus* (se)	Heated with oil	Topical	Skin ulcers
*Chamomilla pubescens* (ap)			Wounds
*Acacia tortilis* (le)	Pit cooked and then mixed with ash and water	Topical	Mange
*Anabasis articulata* (ap)			
*Rhus tripartita* (le)			
Bitumen		Topical	Mange
Exhausted engine oil			
Camel milk			
Camel fat	Mixed and rest for a week	Topical	Mange
Sulphur stone			
*Cistanche phelypaea* (ap)	*Cistanche phelypaea* is cut in pieces, mixed with the other grinded plants and then with bitumen	Topical	Mange
*Hammada scoparia* (ap)			
*Salsola tetrandra* (ap)			
Bitumen			
*Artemisia herba-alba* (ap)	Boiled in water	Topical	Mastitis
*Zygophyllum gaetulum* (ap)			
*Acacia tortilis* (ba)	Grinded and mixed	Topical	Wounds
*Rhus tripartita* (ba)			
*Tamarix* sp. (ba,wo)			

### Changes in knowledge and remedies used

Historically, Sahrawi ethnoveterinary was based on the vegetal, animal, and mineral resources of the customary desert ecosystem of Sahrawi tribes: nomads would harvest or collect the products, process them and apply the remedies and experiment new/different products in accordance to their availability and efficacy. The conceptualization of livestock illnesses was built on nomads’ knowledge of the desert environment and its changes, thus influencing the livestock health and nutritional status. Although analysing and discussing the changes that occurred to Sahrawi ethnoveterinary knowledge around camels is out of the scope of this paper (refer to [24] and [28], respectively, for discussion about the changes that occurred in Sahrawi camel husbandry and knowledge of camel forages with exile, dispossession, and sedentarisation in refugee camps), the Sahrawi knowledge of camel diseases and of their treatments appear to have gone through similar changes in the last decades. There are four main directions of change relating to this study: 1) changes in camels’ pathological states with sedentarisation and the shift from a fully nomadic to a settled and/or semi-nomadic camel husbandry (e.g., changes in nutritional and digestive disorders); 2) changes in the conceptualization of diseases toward a merging of traditional/local conceptualizations and Western explanations, following the exposure of Sahrawi refugees to Western veterinary medicine (e.g., through NGOs cooperation programmes); 3) changes in the remedies used with the incorporation of commercial veterinary drugs (e.g., Ivermectin and Sebacil to treat mange) and other products made available by markets and used in substitution of traditional remedies (e.g., the adoption of the use of bitumen–obtained in Tindouf from road construction enterprises–and exhaust engine oil rather than self-processed vegetal tars in the treatment of mange); and 4) change in the traditional ethnoveterinary knowledge with the disengagement of many Sahrawi from camels and camel husbandry. These processes relate to more general mechanisms of loss and recovery of subsistence practices, including camel husbandry, and of traditional ethnobiological knowledge that are taking place among contemporary Sahrawi [26], and which are relevant also to studies of cultural change and knowledge dynamics of pastoral transitions [91].

## Conclusions

Pastoralists throughout the world have a deep and detailed knowledge of their animals and of local ecosystems, and of the relations intercurring between them. Through centuries of engagement with camels and the desert resources, Sahrawi nomads have developed a system of preventive and applied medicine to maintain or restore the health of their livestock, camels *in primis*. This study provides an account of Sahrawi ethnoveterinary knowledge of camel diseases and their treatments in Western Sahara. The results show how the Sahrawi have historically developed a rich and detailed knowledge of camel illnesses, elaborating strategies to prevent and treat these health conditions. These strategies include movement (e.g., from areas where a disease is present to one where it is not), management (e.g., not letting camels graze early in the morning in presence of dew), and food supplementing (e.g., of salts and bones in absence of halophyte plants in grazing areas). The treatments used are of vegetal, animal, and mineral origin, and largely sourced from the local desert ecosystem. Common administration routes are topical (i.e., applications of plasters or ointments), oral (i.e., feeding and drenching) and by inhalation (i.e., fumigations). Mixtures of several products and cauterization form also part of the therapeutic knowledge of the Sahrawi.

As a whole, these results suggest that 1) knowledge about camel diseases and their prevention and treatment is critical to pastoralists’ success in arid environments, as the survival and reproduction of their herds rely on this knowledge; 2) this knowledge is shaped by and contextualized within the characteristics and constraints of the ecosystem where pastoralists live (i.e., diseases are conceptualized in terms of their environmental relationship, whilst treatments are based on plants and other products from the desert ecosystem); and 3) camel health and diseases are understood as the result of the contraposition of several environmental factors (e.g., dry/wet climate, rocky/sandy soils, presence/absence of certain forage plants, presence/absence of certain disease vectors, etc.). On the basis of these factors, the Sahrawi eventually differentiate between the wet or sandy and halophyte-poor areas at the border of their nomadic territory where some diseases are present (e.g., *homzi* in the north, trypanosomiasis in the south, and *buguashish* in the east), and their customary grazing areas in the riverbeds and rocky plains of Western Sahara and northern Mauritania, where there is dry climate, abundance of *N. perrinii* and other halophyte plants, and absence of trypanosomiasis. These findings enhance our understanding of the critical role of ethnoveterinary knowledge among pastoral populations, which provides a high degree of detail and complexity and is rooted in pastoralists’ understanding of environmental and ecological relationships. In light of the results provided by this study and of the knowledge gap that currently exists on camel diseases, further studies are warranted to source relevant data on the ethnoveterinary practices of camels pastoralists, in order to improve camel management strategies. We suggest that scholars should investigate in more detail the environmental aspects related to camel health and diseases focusing, for example, on the relationships between camels’ nutritional deficits and nomads’ herding strategies.
